# Case Report: Combined Laparoscopic Perineal Hernia and Abdominal Parastomal Hernia Repair With a Mesh After Abdominoperineal Resection: A Video Vignette and Review of the Literature

**DOI:** 10.3389/jaws.2024.13261

**Published:** 2024-11-22

**Authors:** F. Brucchi, C. Limongi, E. Masci, F. De Stefano, E. Pelfini, D. Cassini, G. Clarizia, M. Franzini, G. Faillace

**Affiliations:** ^1^ General Surgery Grad School, University of Milano Statale, Milan, Italy; ^2^ Department of General Surgery, ASST Nord Milano, Milano, Italy; ^3^ Department of General Surgery, ASST Valtellina e Alto Lario, Sondrio, Italy

**Keywords:** incisional hernia, parastomal hernia, laparoscopy, rectal cancer, mesh

## Abstract

**Background:**

Postoperative perineal hernia (PH) is an uncommon complication after abdominoperineal resection (APR). Different techniques have been described in literature and there is no consensus regarding the optimal repair approach. In the present study, we reported a case of a laparoscopic combined repair of a perineal hernia and abdominal parastomal hernia (PSH) with mesh. Studies have shown that the prosthetic PSH and PH repair can be performed at the same time by laparoscopy with the same trocars positioning, adding the advantages of minimally invasive surgery and avoiding large laparotomy.

**Methods:**

A literature search in Pubmed was performed. All articles in English describe laparoscopic repair of combined perineal and parastomal hernias. A case presentation of an 83-year-old woman with combined parastomal and perineal hernias after abdominoperineal resection (APR) shown in a video vignette is provided.

**Results:**

Three single patient case reports published between 2016 and 2023 were found in literature. Two patients with rectal cancer underwent APR procedure, while the third patient underwent an anterior pelvic exenteration (APE) for carcinoma of the urinary bladder (CUB). The laparoscopic procedures did not require conversion and all procedures successfully closed the defect using a mesh. In our case, the operative time was 3 h with the major time spent for PH repair. The intraoperative blood loss was non-significant and the postoperative course was regular. The patient has been discharged on the fourth postoperative day. At 1 year follow-up, the patient noticed a great improvement in her daily-life due to the absence of the previous discomforts and there was no evidence of early recurrence or other postoperative complications.

**Conclusion:**

Combined laparoscopic transabdominal PH and PSH repair with the use of synthetic mesh was shown to be a safe and effective repair for this rare disorder. To accurately compare techniques, we require prospective studies with longer follow up durations.

## Introduction

Abdominoperineal resection (APR) is a procedure performed for low rectal cancer when sphincter preservation is not feasible. The late complications include perineal and parastomal hernias [1].

Perineal Hernia (PH) is defined as a pelvic floor defect through which the intra-abdominal viscera may protrude. PH can be primary (congenital) or secondary (post-operative). The latter generally occurs after pelvic surgery, such as rectal or prostate surgery. PH can be classified as anterior when located anteriorly to the transverse perineal muscle, or posterior, if it occurs in the levator ani muscle or between the levator ani and the coccygeus muscles [2]. The incidence of PH is less than 1%, however, the true incidence could be higher due to the under-reporting and under-detection of asymptomatic PH. The spread of radiotherapy and extralevator resection increases the incidence of PH with rates 12%–26% [3]. Symptoms usually occur within the first two postoperative years. The commonest presentation is a painful bulging mass which causes discomfort when standing or sitting. Complications of PH include urinary dysfunction, skin erosions and bowel obstruction. Large perineal hernias require surgical intervention based on the severity of the complication. Only few studies exist and most of them are case reports with low numbers and short-term follow-up. There are different and multiple approaches (perineal, abdominal, combined and laparoscopic) and techniques (suture, pexy, synthetic and biological mesh or flap) available. As a result, there is no consensus on the optimal management of PH.

Parastomal hernia (PSH) is an incisional hernia located at or immediately adjacent to a stoma, with an incidence that approaches 50% at 2 years postoperatively. In the last decade, different approaches of minimally invasive procedures have been proposed for the treatment. At present, there is no clear superiority of one technique over another. Khritarides et al. showed that the keyhole technique was associated with the highest incidence of postoperative complications and recurrences (31.3% and 24.1%, respectively), followed by the Sugarbaker technique (27.6% and 9%, respectively). The patients undergoing the keyhole technique experienced the shortest cumulative length of hospital stay. Although the meta-analysis published by Kritharides et al. highlights an advantage in terms of recurrence and safety for the new techniques (“sandwich” and “hybrid with 3D mesh”), a tailored surgical approach appears optimal in these cases [4].

Abdominal laparoscopy offers a good solution because both PH and PSH repairs can be performed using the same trocars disposition.

In this study we sought to review the existing literature on fully laparoscopic repair of combined perineal and parastomal hernias, provide a video vignette that illustrates the principal steps and provide practical guidance.

### Materials and Methods

The peer-reviewed literature published up to 29th June 2024 was searched using Medline (PubMed), Embase, Scopus, and Cochrane Library databases with MeSH terms: “perineal hernia repair,” “laparoscopy,” “minimally invasive surgery,” “parastomal hernia repair.” Papers written in English describing repair of combined parastomal and perineal hernias after the removal of the rectum were included. Exclusion criteria were reviews, systematic reviews, meta-analysis, articles about primary or congenital PH, animal studies. A case presentation, in line with the SCARE Criteria [5] at the end of the introductory section, of a patient with combined parastomal and perineal hernias was related through video vignette. The operative details of the case and advice gained from this experience were provided. Written informed consent was obtained from the patient for the publication of this case report.

### Results and Literature Review

Three articles published between 2016 and 2023 were included (Table 1). All three articles were single patient case reports. Two articles document patients with rectal cancer underwent APR procedure, while the third patient underwent an anterior pelvic exenteration (APE) for carcinoma of the urinary bladder (CUB). Two procedures were fully laparoscopic and one was open. The patient positioning was different between papers, opting for Trendelenburg with rightward tilt in the article by Dapri et al. and lithotomy position in the case reports by Shenoy et al. and McDonald et al. The two laparoscopic procedures successfully closed the defect without requiring conversion. None of the patients reported significant perioperative or postoperative complications, and no recurrence was noted in any of them in the 1 year follow-up period.

**TABLE 1:** Table showing the available literature on the topic.

Author	Sample size	Study design	Approach	Indication	Positioning	Mesh
Dapri [1]	1	Case report, video vignette	Laparoscopic	Hx laparoscopic APR	Trendelenburg, tilted right	Surgimesh XB, polypropylene Implant With Silicone Exclusion Barrier
Shenoy [6]	1	Case report	Laparoscopic	Hx open APE, RC, RH, urethrectomy, radical vaginectomy with IC	Lithotomy	Merinium mesh, polypropylene/Polylactide-Caprolactone
McDonald [7]	1	Case report	Open	Hx laparoscopic APR and RH	Dorsal Lithotomy	Gore Dual

## Case Description

An 83-year-old female with a prior history of low rectal cancer (pT2N0M0) underwent a laparoscopic abdominoperineal resection (APR) with permanent colostomy. Adjuvant treatment was not recommended by the multidisciplinary team. A year post-surgery, she presented with symptomatic and extensive perineal hernia coupled with abdominal parastomal hernia. The patient complained of perineal pain with sitting and walking, along with symptoms associated with parastomal hernia, including the need for a manual reduction after coughing.

## Timeline

**FIGURE 1:**
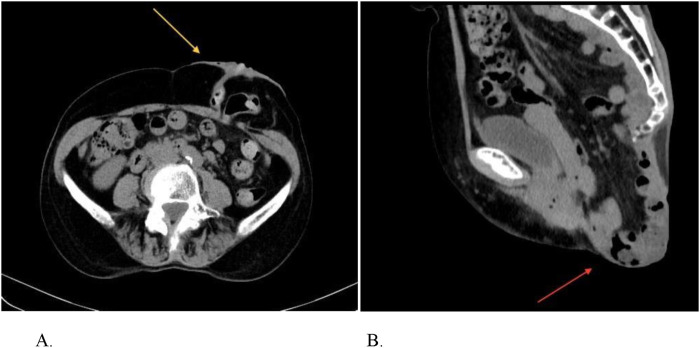
Pre-operative CT abdomen/pelvis showing the hernia defects. **(A)** Parastomal hernia. **(B)** Perineal hernia.

Colonoscopy was performed to rule out organic lesions. Preoperative CT scans revealed no cancer recurrence but showed little parastomal hernia measuring 2.5 cm, classified as a type I according to the 2013 EHS classification [8], in diameter and a perineal hernia measuring 5 cm in diameter, containing small bowel on axial, coronal, and sagittal cross-sections (Figure 1), with minimal uterine-bladder prolapse. She was scheduled for fully laparoscopic pelvic dissection with small bowel reduction, followed by concurrent repair of the perineal and parastomal hernias, as demonstrated in the accompanying video (see Supplementary Video).

**FIGURE 2:**
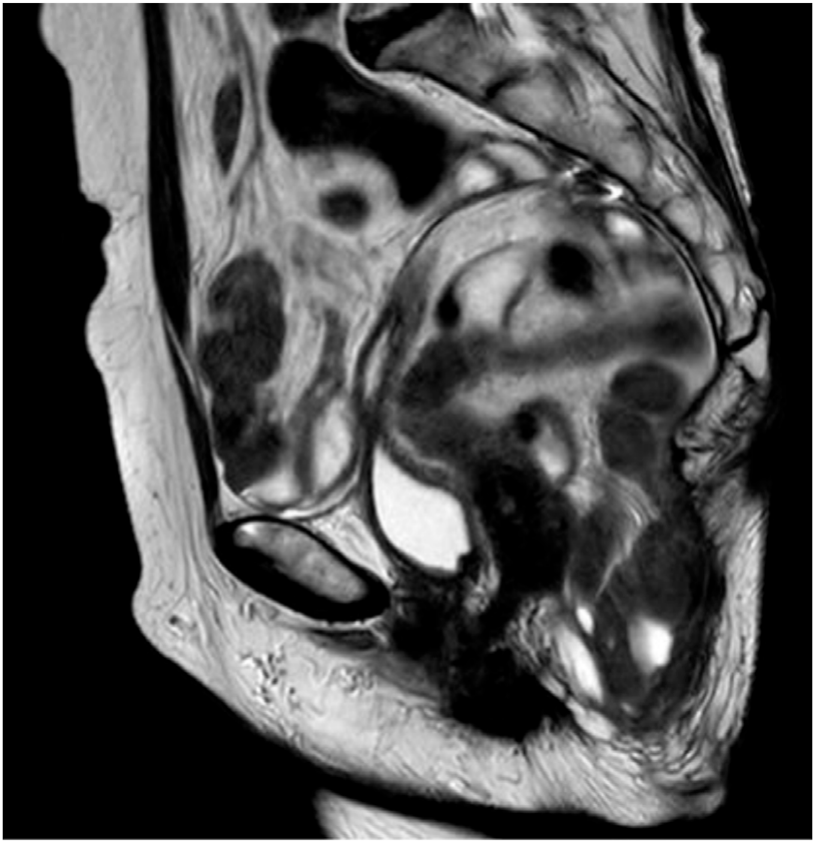
Post-operative MRI pelvis showing no perineal recurrence. Sagittal plane.

An MRI scan without contrast was performed 1 year postoperatively, showing no recurrence, either parastomal or perineal (Figure 2).

## Diagnostic Assessment and Interpretation

The patient was placed in a low lithotomy position with Trendelenburg tilted right, and the pneumoperitoneum was induced in Palmer’s point with Veress needle.

Four trocars were placed respecting the triangulation view: three 12 mm trocars were placed in the right lower quadrant, paraumbilical site and umbilical site, one 5 mm trocar in Palmer’s point. The surgeon and first assistant were stationed on the patient’s right side, the second assistant on the left. There were no intraoperative complications, and the patient was discharged on postoperative day 4 with subcutaneous drainage in the perineal region.

Subsequent follow-ups at 2 months, 6 months, and 1 year revealed no evidence of recurrence, indicating favorable progress.

## Discussion

Due to the uncommon nature of the pathological conditions being addressed, it is apparent that the available literature on the subject is limited, consisting mainly of case reports.

The majority of procedures found are described as one-step repairs performed via laparoscopic or open. Although the data are limited, the absence of reported complications, necessity for conversion, or hernia recurrence, even within the constraints of a restricted follow-up period, suggests that the laparoscopic approach stands as a safe and viable method for repairing these particular hernias.

Parastomal hernia is a rare, underreported, and poorly studied condition with challenging management and no universally accepted treatment algorithm available in the literature [9]. The perineal approach offers enhanced surgical field exposure compared to the abdominal approach, facilitating mesh placement, fixation, and repair of the cutaneous defect resulting from perineal pressure exerted by the PH. Conversely, the abdominal approach allows for easier mobilization of herniated contents into the abdominal cavity and safer adhesiolysis [10]. Additionally, the abdominal approach enables confirmation of the absence of cancer recurrence.

The meta-analysis conducted by Maspero et al. indicated that the abdominal approach may present a lower absolute risk of morbidity and surgical site occurrences (SSOs) compared to the perineal approach, although the findings did not reach statistical significance.

Another debated issue is whether to perform primary repair or use a mesh. This same meta-analysis suggests that there appears to be no significant difference between using a mesh and primary repair; however, despite this, many authors recommend the use of a mesh [9].

In our accompanying video vignette we present our approach to this technique. We opted to position the patient in lithotomy to ease the suturing of the perineal cutaneous flap. Adhesiolysis and removal of migrated viscera from the perineal hernia were conducted abdominally, utilizing the same trocars positioned for the parastomal hernia repair.

The bladder was distended with a physiological solution and Methylthioninium chloride before being completely detached and mobilized anteriorly, towards the uterus. Following the liberation of Cooper’s ligament and the pubic tubercle, the peritoneum was meticulously sutured, juxtaposing the vesical and pelvic peritoneum.

A titanium mesh (TiMESH Strong 30 × 30 cm) previously cut to size was placed to close the perineum defect. A crucial consideration is selecting the optimal sites for mesh anchorage. Our recommendation is to affix the mesh to the periosteum of the sacrum for enhanced strength, anteriorly to the Cooper’s ligament and pubic tuberculum, overlaying the bladder, and laterally to the parietal peritoneum covering the branches of the iliac vessels, splanchnic nerves, and ureters. Intraoperatively, we measured the defect using a soft ruler to ensure proper positioning and adequate overlap on both sides. Subsequently, we securely fastened the mesh using helical tacks to the periosteum both posteriorly and anteriorly. Laterally we used a continuous suture with (FilBloc^®^90) to anchor to the peritoneum alone in order to provoke vessels, nerves or ureters injury. Finally, we utilized fibrin glue to ensure a fixation of the mesh stronger.

The use of this type of mesh, to our knowledge, has been reported in the literature for laparoscopic inguinal hernia and large hiatal hernia [11] repairs, with good results in terms of analgesic use and the sensation of a foreign body [12].

Subsequently, the procedure continued with the PSH repair. Mobilization of the stoma into the defect proved to be very difficult due to the extensive adhesions between the colon and the sac. We proceeded with meticulous dissection to free the colon loop. Noting the short length of the colon loop, we opted to perform a keyhole technique to repair the defect and facilitate a tension-free stoma. Closure of the defect was achieved using a continuous suture with Filbloc^®^. A fenestrated mesh (TiMESH Strong) measuring 15 cm × 15 cm was then placed around the stoma according to the Keyhole technique and securely fixed in place with CapSure™ device.

Various approaches for PSH repair are described in the literature. The most recent meta-analysis suggests that the two most effective and safest techniques, in terms of recurrence rates, are the “sandwich” and “hybrid” approaches with 3D meshes. However, no single technique has proven to be definitively superior to others, and a tailored surgical approach may ultimately be the most effective.

Li Luan et al. developed an algorithm to identify the optimal technique for treating recurrent parastomal hernias. They initially used laparoscopy to assess for infection, adhesions, or tumor recurrence. If infection was present, they performed a simple suture repair. Adhesions were classified as light, medium, or heavy. For light adhesions with a short bowel loop, the keyhole technique was used; with a long bowel loop, the Sugarbaker approach was preferred. Medium adhesions with bowel injury led to onlay mesh repair, while in the absence of bowel injury, laparoscopic redo surgery with or without the keyhole/Sugarbaker technique was utilized. For heavy adhesions, onlay repair was favored. This algorithm resulted in zero recurrences over a mean follow-up of 32.8 ± 3.77 months, covering 17 cases [13].

There is limited literature available which describes combined approach repair of PH and PSH hernias. However, the laparoscopic approach for combined parastomal and perineal hernia repair seems a safe and feasible technique, providing a viable alternative to open and perineal methods. However, further research and data collection are necessary to determine any differences compared to other approaches.

## Patient Perspective

One year after the surgery, the patient reports being satisfied, experiencing no symptoms, and has resumed normal daily activities.

## Data Availability

The original contributions presented in the study are included in the article/Supplementary Material, further inquiries can be directed to the corresponding author.
